# Impact of the COVID-19 pandemic on obstetrical care at a tertiary care facility in Mombasa, Kenya: Time-series analyses and staff perspectives

**DOI:** 10.1371/journal.pgph.0001829

**Published:** 2023-04-19

**Authors:** Jessica E. Long, George Wanje, Nawal Aliyan, Dickson Okello, Barbra A. Richardson, Nyambura Wanjiru-Korir, Khadija Shikely, Aisha Abubakar, Pauline Oginga, R. Scott McClelland

**Affiliations:** 1 Department of Epidemiology, University of Washington, Seattle, Washington, United States of America; 2 Department of Medicine, University of Washington, Seattle, Washington, United States of America; 3 Department of Medical Microbiology, University of Nairobi, Nairobi, Kenya; 4 Department of Global Health, University of Washington, Seattle, Washington, United States of America; 5 Coast General Teaching and Referral Hospital, Mombasa, Kenya; 6 Department of Biostatistics, University of Washington, Seattle, Washington, United States of America; 7 Department of Health Services, County Government of Mombasa, Mombasa, Kenya; University of Embu, KENYA

## Abstract

The COVID-19 pandemic caused disruption in healthcare delivery due to reductions in both health facility capacity and care-seeking behavior. For women experiencing obstetric complications, access to comprehensive emergency obstetric care is critical for maternal and child health. In Kenya, pandemic-related restrictions began in March 2020 and were compounded by a healthcare worker strike in December 2020. We examined medical record data at Coast General Teaching and Referral Hospital, a large public hospital, and conducted staff interviews to understand how healthcare disruptions impacted care delivery and perinatal outcomes. Routinely collected data from all mother-baby dyads admitted to the Labor and Delivery Ward from January 2019 through March 2021 were included in interrupted time-series analyses. Outcomes included number of admissions and proportion of deliveries that resulted in caesarean sections and adverse birth outcomes. Interviews were conducted with nurses and medical officers to understand how the pandemic impacted clinical care. Pre-pandemic, the ward averaged 810 admissions/month, compared to 492 admissions/month post-pandemic (average monthly decrease: 24.9 admissions; 95% CI: -48.0, -1.8). The proportion of stillbirths increased 0.3% per month during the pandemic compared to the pre-pandemic period (95% CI: 0.1, 0.4). No significant differences were seen in the proportion of other adverse obstetrical outcomes. Interview results suggested that pandemic-related disruptions included reduced access to surgical theaters and protective equipment, and absence of COVID-19 guidelines. While these disruptions were perceived as impacting care for high-risk pregnancies, providers believed that overall quality of care did not diminish during the pandemic. However, they expressed concern about a likely increase in at-home births. In conclusion, while the pandemic had minimal adverse impact on hospital-based obstetrical outcomes, it reduced the number of patients able to access care. Emergency preparedness guidelines and public health messaging promoting timely obstetrical care are needed to ensure continuation of services during future healthcare disruptions.

## Introduction

The spread of the severe acute respiratory syndrome coronavirus 2 (SARS-CoV-2) and resulting COVID-19 (coronavirus disease 2019) pandemic had far-reaching consequences beyond the immediate morbidity and mortality associated with COVID-19 illness. One such harm was the disruption in healthcare delivery due to both decreases in care seeking behavior and reduced capacity of health facilities to provide care. Beginning in early 2020, a myriad of factors impacted care seeking behavior, including reduced access to transportation, changes in childcare responsibilities, government policies and mitigation efforts that restricted movement and travel, and fear of contracting SARS-CoV-2 [1–4]. Within healthcare systems, changes in infrastructure and staffing, safety measures to protect against COVID-19 transmission, and staff burnout all contributed to a reduced capacity to provide care [5, 6].

For women experiencing complications during the pre- and perinatal period, access to comprehensive emergency obstetric and neonatal care (CEmONC) can be critical for survival of both mother and infant. CEmONC facilities provide both standard birth monitoring and emergency care, such as caesarean sections, to treat obstetrical complications and prevent maternal mortality and fetal demise [7–12]. Evaluations of previous large-scale CEmONC interruptions, such as during the 2014 Ebola epidemic, suggest that decreases in use of vital healthcare services contribute to increases in stillbirths [13]. To date, studies examining the impact of the COVID-19 pandemic on obstetrical care delivery and adverse birth outcomes have primarily focused on high-income countries (HICs) and have found mixed results. In HICs, some studies have found an increase in stillbirth [14–16], but most studies have found no adverse impact of the pandemic. A meta-analysis of 12 studies in HICs found a reduction in preterm birth following the onset of the pandemic [15]. Fewer studies have examined outcomes in low-and middle-income countries (LMICs), with most evidence coming from Southeast Asia and very few studies from LMICs in Africa [14, 17, 18]. The evidence to date suggests that LMICs may have had a greater adverse impact on obstetrical outcomes as a result of the pandemic, including increases in stillbirths and maternal mortality [15].

In Kenya, care disruptions due to the COVID-19 pandemic began in March 2020, and were further compounded by a healthcare worker strike beginning in late 2020. The combined disruptions likely impacted both the care provided in health facilities and the ability of women to seek labor and delivery care. The aim of this analysis was to understand the impact these disruptions had on care delivery and birth outcomes at the third largest public tertiary care facility providing CEmONC services in Kenya.

## Methods

### Study design and setting

This multi-method study combined a single-site retrospective quasi-experimental study using routinely collected medical record data and staff interviews from Coast General Teaching and Referral Hospital (CGTRH) in Mombasa, Kenya. As the largest tertiary care facility in the coastal region, CGTRH is the referral hospital for approximately 1.1 million people in Mombasa County, as well as nearly 2 million people in surrounding counties. As of the 2014 Kenyan Demographic and Health Survey, 82% of infants in Mombasa County were delivered in a healthcare facility, with 59% delivering at public sector facilities and 23% at private facilities [19].

Prior to the COVID-19 pandemic, the CGTRH Labor and Delivery Ward had 16 beds, two surgical theaters and an 8-bed High Dependency Unit (HDU) to provide more intensive care for women experiencing complications during labor. Clinical care within the Labor and Delivery Ward is led by consultants (doctors with advanced clinical degrees in Obstetrics and Gynecology and Master of Medicine degrees in their respective fields), assisted by a team of medical officers (MOs), MO interns, and nurses. From 2015–2019, approximately 9,900 women were admitted to the CGTRH Labor and Delivery Ward each year (data from internal hospital records). As the primary referral facility, CGTRH receives a higher proportion of women admitted with pregnancy complications compared to other care facilities in Mombasa County.

The first COVID-19 cases were identified in Kenya on March 13^th^, 2020, and the government began instituting restrictions on March 18^th^. Specific measures included curfews, travel restrictions, school closures, bans on gatherings of >15 people, and drastic restrictions on public transportation. Mask mandates required use of face masks in public, people were required to maintain 1.5 meters physical distance in public and workspaces, and hand washing and use of hand sanitizers were required. Despite these measures, case numbers continued to grow. Early in the pandemic, Mombasa County was among the counties with the highest numbers of cases in Kenya and implemented a full lockdown in some areas on 6^th^ May 2020. During the first year of the pandemic, from March 2020 to March 2021, 9,915 COVID-19 cases were documented in Mombasa County [20]. CGTRH served as the main treatment center for severe and critical cases of COVID-19 in the coastal region. As a result, the Maternity Unit, including the Labor and Delivery Ward, was adapted to accommodate a 130-bed COVID-19 isolation unit, and maternity services were relocated to other areas of the hospital.

In addition to the pandemic, care delivery was interrupted in Mombasa County due to a national public sector healthcare worker strike. In October and November 2020, healthcare workers in Mombasa began a “go-slow” period in which healthcare services were restricted to emergencies, followed by a full strike from December 28, 2020 –February 19, 2021. These events resulted in temporary closure of some public health facilities and reduced staffing at others, including CGTRH. The strike was primarily due to delays in pay.

### Study population

Quantitative analysis in this study included all patients admitted to the CGTRH Labor and Delivery Ward from January 2019 through March 2021. Interviews were conducted among CGTRH MOs and nurses to gain insight into how the COVID-19 pandemic influenced the patient population seeking care and the services provided in the Labor and Delivery Ward.

### Data collection

Quantitative data were abstracted from routinely collected medical record data. The Labor and Delivery Ward at CGTRH aggregates summary data into daily counts of admissions and birth outcomes, which are saved in an electronic Maternal Registry. The Maternal Registry is maintained on a Microsoft Excel (Redmond, WA, USA) spreadsheet by the Health Records and Information Officers at CGTRH. For this analysis, all aggregated Maternal Registry data for admissions during the analysis period were obtained. No identifying information of individual Labor and Delivery Ward patients was collected. Qualitative data collection included semi-structured interviews and input from key stakeholders. Interviews with hospital staff were conducted in person or by phone and were audio recorded, with the option to decline recording.

### Outcomes

For quantitative analyses, the variables of interest were indicators of care delivery and adverse birth outcomes. Care delivery indicators included overall admission count and proportion of deliveries that were caesarean sections. Adverse birth outcomes included proportion of deliveries that were premature (defined as <37 weeks gestational age), stillbirths (defined as fetal death after 28 weeks gestation), and maternal mortalities. These quantitative outcomes were then contextualized using the interview data obtained from hospital staff. The interviews aimed to assess how the COVID-19 pandemic impacted care-seeking behavior among patients, the types of patients that were admitted, and the ability of the CGTRH clinical team to provide care. Interviews also assessed the ways in which hospital staff believed the pandemic impacted birth outcomes overall in Mombasa.

### Statistical analyses

The impact of the COVID-19 pandemic on care delivery and obstetrical outcomes at CGTRH was assessed using pre-post comparisons as well as a sequence of interrupted time-series models with segmented regression. A pre-post comparison of admissions was examined using t-tests for differences in means before and after pandemic onset. Pre-post comparisons of all proportion outcomes were examined using means and linear regression weighted by number of deliveries per month. For interrupted time-series analyses, each outcome of interest was modeled separately using the same modeling technique. Two time segments were modeled; the pre-pandemic period (January 2019 –February 2020) and the pandemic period (March 2020 –March 2021). Segmented ordinary least-squares regression with Newey-West standard errors was used to account for autocorrelation. The following model was used for all interrupted time-series regressions:

$$Y_{t}=B_{0}+B_{1}T_{t}+B_{2}X_{t}+B_{3}X_{t}T_{t}+B_{m}Month+e_{t}$$

Where Y_t_ represents the outcome at each monthly time point *t*, B_0_ estimates the baseline level of each outcome of interest at the start of the study period, B_1_ estimates the average monthly change in each outcome, T_t_ is month since study start, B_2_ estimates level changes that occurred right when the pandemic began, X_t_ indicates the pandemic period, and B_3_ estimates the difference in monthly trends during the pandemic compared to the pre-pandemic period [21]. An additional parameter, B_m_, estimates the outcome for each calendar month (coded 1–12), to account for seasonal changes unrelated to the pandemic. Differences in linear trends were considered significant at p<0.05. We assessed for autocorrelation using the Durbin-Watson statistic [22] and weighted proportion outcomes by number of monthly deliveries. Sensitivity analyses were conducted repeating all interrupted time series analyses while controlling for the period that healthcare workers were on strike. All analyses were conducted using Stata v15.1 (College Station, TX, USA).

Qualitative data were analyzed using a framework method approach [23]. Data included semi-structured interviews with hospital staff as well as input from key stakeholders including the lead consultant at the Labor and Delivery Ward who is an investigator on this project (NA). MO and nursing staff in the Labor and Delivery Ward at CGTRH were informed of the research and were invited to interview. Interviews were completed with all MOs (n = 3) and with nursing staff until saturation in key themes had been reached. Selection of nurses was determined based on inclusion of nurses from different cadres and availability of nursing staff. Interviews followed a topic guide outlining key themes and were recorded through field notes and audio recordings (GW, DO) and transcribed. Transcripts were reviewed by two coders (GW, JL). A structured codebook was created in Microsoft Excel using an initial set of codes that were derived using the interview guide, then were adapted to include new themes that emerged during the interviews. The codebook was populated by the coders using field notes and audio transcripts. Disagreements in coding were discussed by the coders until consensus was reached. Interview results were also reviewed with key stakeholders at CGTRH Labor and Delivery Ward and the Mombasa County DOHS to assess agreement with interview findings.

### Ethics statement

All study procedures were approved by the Kenyatta National Hospital—University of Nairobi Ethics and Research Committee, the CGTRH Ethics Review Committee, and the University of Washington Human Subjects Division Institutional Review Board. Aggregate outcome data did not require informed consent as no identifying patient data were collected. Written informed consent was obtained from all interview participants. All interview data were saved on password protected computers and consent forms were locked in secure filing cabinets. All interviews were conducted by members of the research team in Mombasa (GW, DO), who are members of the Mombasa community and had established a working relationship with the CGTRH Labor and Delivery Ward staff prior to the interviews. Interviews were overseen by the qualitative research lead (GW) who has extensive experience conducting qualitive research related to women’s health in Mombasa County. The study team also includes members of the Mombasa County DOHS (KS, PO, AA), as well as the lead consultant of the Labor and Delivery Ward (NA), who provided key insight into context within Mombasa County and the Labor and Delivery Ward during the study. These team members contributed to study design and also served as key stakeholders, contributing knowledge gained from their roles in the community.

## Results

### Health care delivery

**Fig 1:**
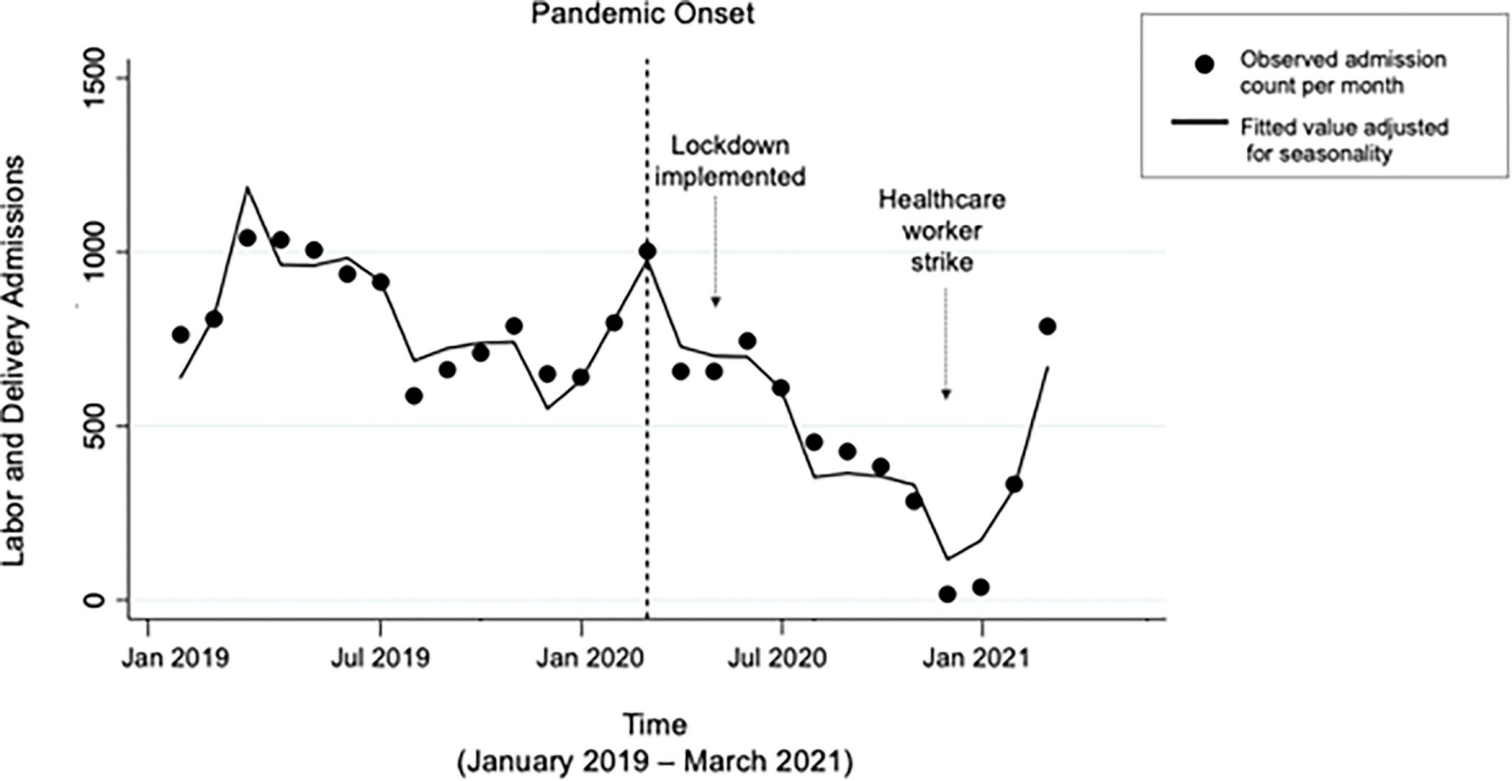
Counts of admissions comparing pre-pandemic (January 2019 –February 2020) to pandemic (March 2020 –March 2021) periods.

From January 2019 through March 2021, CGTRH had 17,734 admissions to the Labor and Delivery Ward, with an average of 810 admissions per month prior to the COVID-19 pandemic and 492 per month after the onset of the pandemic (p = 0.0013). Table 1 and Fig 1 demonstrate trends in health care delivery before and during the pandemic. Relative to the pre-pandemic period, admissions at CGTRH dropped by an average of 25 admissions per month (95% confidence interval [CI]: -48, -1.8; p = 0.037) after the onset of the pandemic.

**Table 1:** Service delivery and adverse birth outcomes comparing the pre-pandemic (January 2019 –February 2020) and pandemic (March 2020 –March 2021) periods, as well as the change in monthly trend post-pandemic onset.

Outcome	Pre-pandemic		During pandemic			Monthly trend post-pandemic onset		
**Total count**	Monthly Mean	95% CI	Monthly Mean	95% CI	p-value^1^	Estimate	95% CI	p-value^2^
Admissions	810	(721, 899)	492	(318, 666)	0.0013	-24.9	(-48.0, -1.8)	0.037
**Proportion of all deliveries**	Proportion	95% CI	Proportion	95% CI	p-value	Estimate	95% CI	p-value
Caesarean section	27.4%	(25.2, 29.6)	33.5%	(31.1, 35.8)	0.001	-0.7%	(-1.5, 0.2)	0.115
Premature	4.4%	(3.5, 5.3)	5.2%	(3.6, 6.9)	0.343	0.0%	(-0.4, 0.3)	0.828
Stillbirth	3.7%	(2.9, 4.4)	4.5%	(3.6, 5.3)	0.173	0.3%	(0.1, 0.4)	0.005
Maternal mortality	0.6%	(0.5, 0.7)	0.7%	(0.3, 1.2)	0.443	0.0%	(-0.1, 0.0)	0.694

^1^Pre-post comparisons were examined using t-tests for differences in means for counts, and linear regression weighted by the total number of month deliveries for proportions (α = 0.05).

^2^Segmented ordinary least-squares regression with Newey-West standard errors was used to estimate monthly trends after the onset of the pandemic.

**Fig 2:**
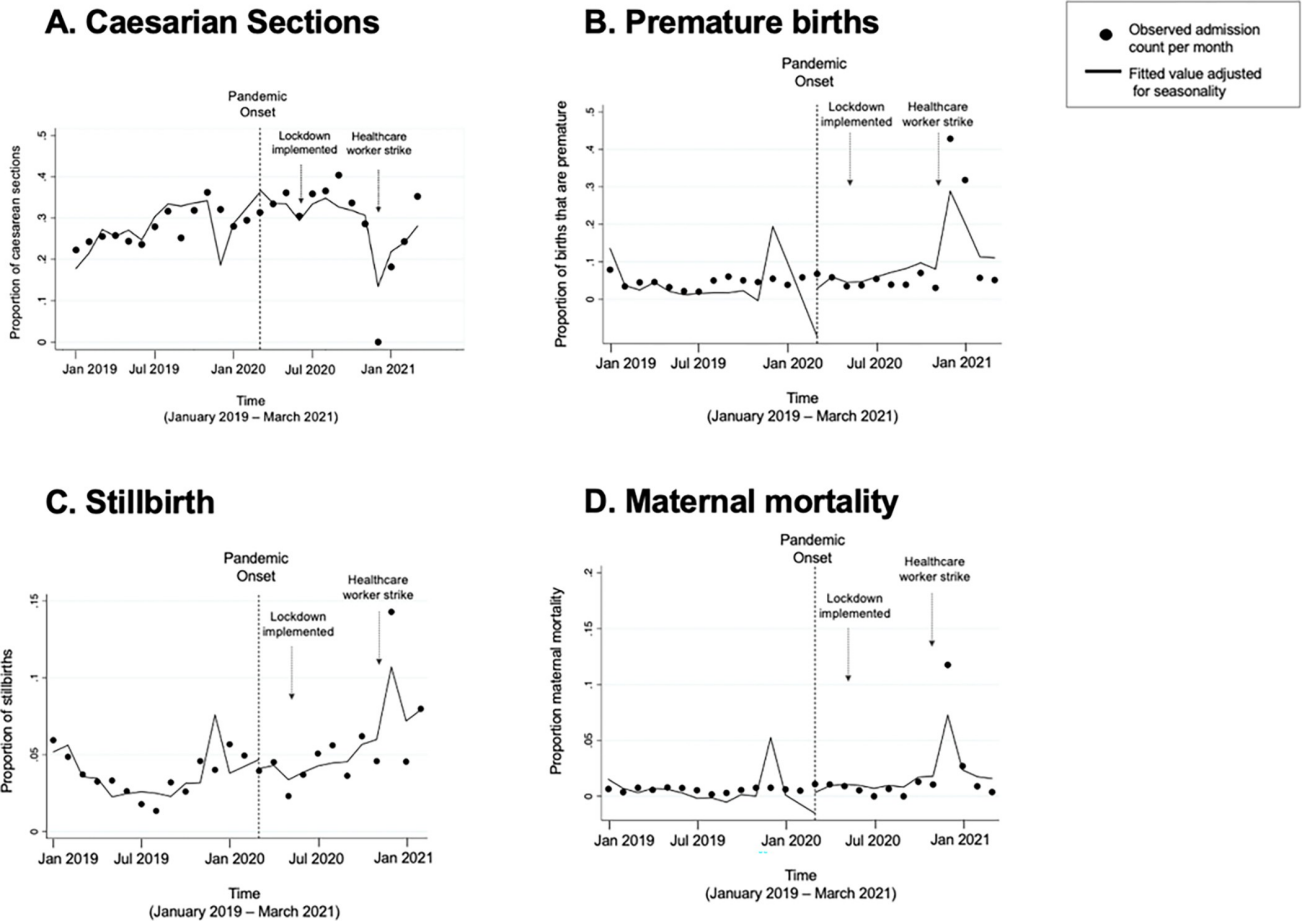
Proportion of total admissions resulting in caesarean sections, premature births, stillbirths, and maternal deaths comparing pre-pandemic (January 2019 –February 2020) to pandemic (March 2020 –March 2021) periods.

Compared to the pre-pandemic period, after the onset of the pandemic the proportion of births via caesarean section increased slightly, from 27.4% of births prior to the pandemic, to 33.5% after pandemic onset (p = 0.001). However, time series analyses controlling for seasonality did not indicate a significant change in proportion of caesarean sections during the post-pandemic period relative to the pre-pandemic period (estimate -1.7%; 95% CI -1.5, 0.2; p = 0.115) (Fig 2).

### Adverse birth outcomes

Prior to the pandemic, 4.4% of all births were premature, compared to 5.2% after pandemic onset (p = 0.343), and the temporal trends in premature births after the pandemic onset did not suggest a significant change compared to the pre-pandemic period. There was a slight increase in the proportion of births resulting in stillbirth during the pandemic. Stillbirths occurred in 3.7% of births in the pre-pandemic period compared to 4.5% during the pandemic (p = 0.173). In the interrupted time-series analysis, this represented a monthly increase of 0.3% (95% CI 0.1, 0.4; p = 0.005) during the pandemic compared to pre-pandemic (Fig 2). Finally, maternal mortality occurred in 0.6% of births prior to the pandemic compared to 0.7% of births during the pandemic (p = 0.443), and analysis of trends did not suggest a significant change after pandemic onset.

To examine the impact the healthcare workers strike had on outcomes, analyses of post pandemic-onset trends, relative to pre-pandemic, were repeated controlling for the strike (Table 2). Figs 1 and 2 demonstrate how the strike period (December 2020 –February 2021) resulted in outliers for all health care delivery and outcome measures. For both admissions and proportion of stillbirths, the trends observed in primary analyses were attenuated when the strike was controlled for and were no longer statistically significant.

**Table 2:** Change in monthly trend in service delivery and adverse birth outcomes in the period post pandemic onset, controlling for the strike (December 2020 –February 2021).

Outcome	Monthly trend post-pandemic onset		
**Total count**	Estimate	95% CI	p-value^1^
Admissions	-12.5	(-35.1, 10.2)	0.247
**Proportion of all deliveries**	Estimate	95% CI	p-value^1^
Caesarean section	-0.4%	(-1.1, 0.4)	0.275
Premature	-0.1%	(-0.5, 0.3)	0.569
Stillbirth	0.0%	(-0.1, 0.2)	0.291
Maternal mortality	0.0%	(-0.1, 0.0)	0.103

^1^Segmented ordinary least-squares regression with Newey-West standard errors was used to estimate monthly trends after the onset of the pandemic.

### Staff perspectives

Interviews were completed with a total of 10 clinical staff at CGTRH between September and December 2021. Of these, three were conducted with MOs and seven with nurses. The median time that the staff member had worked in the Labor and Delivery Ward at CGTRH was 3 years, with a range of 1 to 15 years. Primary themes of the interviews included the changes to Labor and Delivery Ward infrastructure and resources, care delivery, and outcomes as a result of the pandemic, and how the pandemic impacted the patient population presenting to the Labor and Delivery Ward.

Several staff members discussed how structural changes in the hospital impacted their work during the pandemic. Like many hospitals globally, CGTRH had to rapidly respond when the pandemic began. As the third largest tertiary care facility in Kenya and the primary tertiary care facility in the coastal region, CGTRH needed to provide care for a large number of patients with severe illness due to COVID-19. To serve this influx of new patients, the Labor and Delivery Ward was converted into a COVID-19 ward, and the Labor and Delivery care team shifted to a different part of the hospital. The HDU, which provided intensive care for women experiencing obstetrical emergencies, was converted to a COVID-19 unit. While these changes allowed the hospital to provide intensive care to COVID-19 patients, they caused initial disruptions to workflow in the Labor and Delivery Ward:

“COVID really affected this area. First of all, I’ve talked of that HDU Ward. It was taken by COVID, it is now a COVID Ward. We no longer have HDU in the labor ward.” Nurse #4

“When COVID hit, we were shifted from our area and taken to another place. At that time COVID patients were so many so they needed more beds, so we were shifted. That brought so much confusion. There was a lot of time when people had to get used to the new place and where things are, so time was wasted.” MO #3

The pandemic also interrupted the use of surgical theaters to conduct caesarean sections. In the Labor and Delivery Ward, staff had access to two surgical theaters. After the Labor and Delivery Ward was moved to another part of the hospital, caesarean sections had to be performed in the main theater, which is shared by other wards, including the Emergency Department. Even after the Labor and Delivery staff were moved back to their original ward, only one Labor and Delivery theater was in operation. As a result, the Labor and Delivery team had to use the main theater anytime the Labor and Delivery theater was not available. Further, clinicians needed to bring their own staff to use the theater, which required coordination of a team of nurses, anesthetists, and aides (referred to as “casual workers”) as well as the equipment and medication needed for the surgery. This had an impact on the time required to perform a cesarean section, and placed an added burden on the staff:

“It takes so much time to move cases to main theater. They may be having another case they need to finish, and also the distance is long between [the] Labor and Delivery Ward and main theater. … You as the MO have to move with your staff … from one theater to the other. And it takes so much time. You have to move with all your staff, all your instruments, your casual worker. The whole theater moves to the other theater.” MO #2

The time required to prepare an operating theatre for cesarean section was also impacted when Labor and Delivery Ward patients had SARS-CoV-2 infections. When a patient in the ward had COVID-19, any space she used required fumigation, which could further delay use of the surgical theater. Even when the operating theatre was not needed, patient care was more complicated if a patient tested positive for SARS-CoV-2 at the time of admission to Labor and Delivery:

“A mother walks in and sits with other mothers, she waits with other mothers, then she gets a positive result. … Now we have to come out of the room, the place has to be fumigated, it delays care, causes so much confusion.” MO #2

These issues were exacerbated by variable success in the creation and dissemination of guidelines at the outset of the pandemic. For nursing staff, guidance on how to prevent disease spread were issued promptly. Nurses were provided with training in appropriate use of personal protective equipment (PPE), handwashing, medical waste management, and masking, and they enforced these behavioral interventions within the ward. For MOs, guidelines on how to respond to changes in hospital infrastructure and workflows were sometimes less clear:

“We don’t have a standard protocol for how to do these COVID cases. Like, where should the patient be triaged? If the patient is admitted, who should bring the patient? Is it the COVID isolation team, or is it the Labor Ward team, the theater team [who needs to] go pick up the patient? What happens after that? …There is always so much confusion.” MO #2

“Initially no [there were no guidelines]. There was so much confusion at the clinic. Then they were developed. But at the beginning, we were just working blindly, trying to figure out what to do. Usually if a mother turned positive, that was dramatic. It was so hard, initially.” MO #3

As experienced in many hospitals globally, adherence to guidelines was sometimes hindered by supply chain issues. In some instances, lack of supplies, particularly PPE, made it difficult for staff to properly follow guidelines. Nurses, in particular, discussed how they sometimes lacked supplies. Some expressed concern that this impacted patient care, and frustration about supply issues impacted their ability to follow guidelines:

“Facilities, gowns, gloves, the kinds of things that you are supposed to wear when you attend to COVID [patients], they have not been up to date. To some midwives, there was that fear of attending to such a mother [with COVID], … because they don’t have that equipment. Or sometimes [you] find a client feels she is neglected, so there is that fear that I am not well equipped to attend to such a mother who is suspected COVID or confirmed COVID. It becomes a problem.” Nurse #1

“Yes, we were trained but we were not given PPEs, so it doesn’t help. If you’re training for how to live with COVID and you are supposed to use PPEs and you are not providing PPEs, then it doesn’t make sense.” Nurse #6

Despite the complications that the pandemic introduced, the clinical staff generally did not feel that patient care or outcomes were meaningfully worsened during the pandemic. This preservation of the quality of care for women admitted to the Labor and Delivery Ward was attributed to having fewer patients during the early stages of the pandemic:

“Workload generally decreased during that time, so bad outcomes did not increase.” MO #1

The clinical team cited several factors that contributed to the decrease in patients. First, due to the reduced number of beds and the need to prevent overcrowding, the hospital actively sought to reduce admissions to protect both staff and patients with greater need, including by redefining how far into active labor a woman must be in order to be admitted. In addition, staff believed that fewer women sought care at the hospital, or sought care at a later stage in labor, due to trouble with transportation and fear of COVID-19 exposure at the hospital.

“At that time, admissions were being minimized, so active phase of labor was more than 6cms [cervical dilation] as compared to 4cm. And because of that, and because of lack of transport, patients staying too far, they end up giving birth at home. And that would pose significant risk on the outcome.” MO #1

“The patients were fewer during that time. They feared coming to the hospital, because of COVID, they feared contracting COVID in the hospital. I think they preferred going to the TBAs [traditional birth attendants]. Also, people traveled upcountry, another reason why the number reduced.” Nurse #6

The consensus among staff was that quality of care at CGTRH did not decrease during the pandemic. However, there was some concern that the delays in seeking care because of fear of contracting COVID-19 led to poor outcomes in some women.

“Mothers like staying at home and coming to the hospital late. With COVID it has made it even worse. … They stay at home longer because … they associate the facility with corona. Because there is a COVID center that is attached to our ward. So, you see, it makes the mothers avoid coming to the facility. In a normal situation if a mother knows that she is high risk pregnancy, they know here is where they will get the best care, there is everything, an ICU and everything. Here in this situation, because of COVID, they avoid coming. By the time the mother comes then sometimes it’s too late because they waited too long.” Nurse #5

Some staff speculated that the reduced number of women seen at CGTRH meant an increase in non-medically attended deliveries occurring outside the hospital, or births attended by traditional birth attendants, and that this may have resulted in an increase in adverse outcomes in the community as a whole:

“People stopped getting medical services, stopped even going to clinics, so complications arose… The same things that were contributing to stillbirth before are still the same problems. One difference [is that] COVID made people not go for medical services. People were opting to go for traditional birth attendants and stuff like that.” MO #3

## Discussion

The COVID-19 pandemic and subsequent restrictions led to a sharp decrease in Labor and Delivery Ward admissions at the largest tertiary care facility in the coastal region of Kenya. The low admissions continued throughout the first year of the pandemic, culminating in the lowest rates during a concurrent healthcare worker strike. During the first year of the pandemic, no major differences were seen in the proportion of deliveries by caesarean section or in obstetrical outcomes, though a small upward trend in the proportion of births resulting in stillbirth was observed after the pandemic onset. Interviews with clinical staff suggested that the pandemic caused a great deal of disruption to their work, including reduced access to surgical theaters and closing of their HDU to accommodate COVID-19 patients. The drop in admissions observed during the pandemic was believed to be due to both reduced care-seeking by patients and changes in admission criteria. These changes were perceived as impacting care for high-risk pregnancies. Despite these challenges, the providers overall believed that the quality of care for women admitted to Labor and Delivery was maintained during the pandemic.

Evidence of the global impact of the COVID-19 pandemic on perinatal health outcomes has been mixed. In high-income settings, some studies have suggested that pandemic responses have led to a decrease in preterm birth, likely due to reduced work and movement among women during late stages of pregnancy [14, 15, 24]. Few other adverse outcomes appear to have been strongly impacted by the pandemic in HICs [15]. While studies in the United Kingdom [25] and Italy [26] show an increase in stillbirths, the majority of studies in HICs found no significant differences [14, 15, 27].

In contrast, modelling studies suggest that the impact of the pandemic may be large in LMICs [28, 29]. One model estimated that, due to interruptions in service during the pandemic, an estimated 60,000–200,000 additional stillbirths could occur globally over a 12-month period [29]. These estimates include births occurring outside of hospital settings. In our hospital-based study, we found a small increase in proportion of stillbirths after pandemic onset, which could indicate a greater proportion of high-risk deliveries presenting at the hospital. However, the increase appeared to be largely driven by the healthcare worker strike. When the strike was controlled for, the association suggesting a higher risk of stillbirth after pandemic onset was no longer seen. Meta-analyses of the limited available data from LMICs suggest an overall increase in stillbirth when examining hospital-based births [15]. However, these reviews rely mainly on data from India and Nepal, and the few studies from African countries have reported mixed results [15, 17, 18, 30]. While one study in Uganda found that pandemic lockdowns resulted in decreases in antenatal clinic attendance and increases in hospital-based stillbirths [17], no changes were found at a hospital in Zimbabwe [30], and no increase in maternal or neonatal death was found in national registry data in Sierra Leone [31]. Finally, a national surveillance study of hospital-based births in Botswana found a reduction in adverse perinatal outcomes during COVID-19 [18]. A potential explanation for the lack of strong evidence could be a failure to capture births occurring outside of hospital settings. Both the decrease in admissions at CGTRH and the concerns expressed by staff tend to support the idea that more births were occurring outside of the hospital, posing an increased risk of adverse birth outcomes in the community. Population-level data that includes outcomes of home births would provide additional evidence of the impact of the pandemic on birth outcomes in LMICs. However, these data are difficult to collect, limiting researchers’ ability to directly measure birth outcomes in all women.

While no other pandemic of this magnitude has occurred in modern history, lessons can be drawn from regional responses to the 2014 West Africa Ebola outbreak [32]. Interruptions in care led to significant decreases in antenatal care visits, facility-based deliveries, and caesarean deliveries in Sierra Leone, Guinea, and Liberia [13, 33–36]. Decreases were noted in both supply of healthcare and demand for health services. This decrease in demand was in part due to the belief that health facilities were a source of Ebola infection [13], which mirrors the concerns expressed by CGTRH Labor and Delivery staff that pregnant patients avoided the hospital due to the COVID-19 unit. In Guinea, facility-based deliveries had been increasing prior to the Ebola epidemic, but did not regain these increases even after the epidemic, causing a sustained decline in indicators of maternal and child health [13]. Downstream impacts of reduced availability and utilization of CEmONC services are difficult to quantify, but one model in Sierra Leone estimated an excess of >3,600 adverse events over a one-year period, including maternal deaths, stillbirths, and neonatal deaths [37].

The results of this study add to the body of literature that can be used to inform recommendations for maintaining CEmONC services in the event of widescale healthcare disruptions. First, as with many other facilities globally, the onset of the pandemic led CGTRH to reallocate resources and space to a COVID-19 unit, leading to interruptions in Labor and Delivery service and reduced capacity to provide care. Developing facility-wide pandemic preparedness plans could help mitigate disruptions caused by these changes early in the COVID-19 pandemic. Second, lack of guidelines was cited by several staff members as a limiting factor in providing care. Development of specific guidelines on how to triage patients in the event of a pandemic could minimize delays in care delivery. Third, clinical staff suggested that patients faced numerous barriers in seeking care, including fear of COVID-19 exposure at the hospital. Both delays in admission and home deliveries might be reduced if there was improved community education to dispel misinformation about risk of exposure in hospitals and to emphasize the importance of medical assistance during delivery.

This study had several strengths. We collected all Labor and Delivery Ward admission data from the third largest public tertiary care in Kenya, providing a large dataset of health outcomes spanning pre- and post-pandemic onset. The interrupted time series design provided trends in obstetrical outcomes over time, which allowed for a more nuanced understanding of the impact of the pandemic after controlling for seasonality and the healthcare worker strike. Involvement of key stakeholders (NA, NWK, KS, AA, PO) in design, analysis, and interpretation of the data provided important context about the impact of the pandemic both in Mombasa County and at CGTRH. In addition, interviews with healthcare providers offered explanatory data that was helpful in interpreting the quantitative findings.

This study also had several limitations. Analyses included only aggregated data, so it was not possible to examine individual predictors of adverse outcomes or key indicators of healthcare delivery, such as waiting time before being admitted and receiving care. Further, the data did not include information about COVID-19 infections in women who delivered at CGTRH, so it was not possible to evaluate how individual patients’ COVID-19 illness directly impacted their outcomes. While quality of data reporting at CGTRH is high, it is possible that data were underreported during the strike due to understaffing; however, this was accounted for by controlling for the strike period in the second analysis. Additionally, responses of staff to interview questions about processes in the Labor and Delivery Ward during the first year of the COVID pandemic may have been shaped in part by more general anxiety experienced by medical staff during the pandemic. Regardless, these responses provide key insights into the staff’s perceptions of their experiences during the disruptions caused by the pandemic. Finally, this study only included data from one large public tertiary care facility. While this limits comparison to small facilities, it likely reflects experiences that may be similar across large facilities in LMIC settings in Africa.

## Conclusions

In conclusion, these data suggest that while the pandemic had minimal adverse impact on obstetrical outcomes for women admitted to Labor and Delivery at CGTRH, it reduced the number of patients able to access care, possibly resulting in an increase in home births that carry greater risk of adverse outcomes. The results of this study highlight the need for guidelines on emergency preparedness to optimize the delivery of CEmONC services during future healthcare disruptions, as well as public health messaging to ensure that women continue to seek timely obstetrical care.

## Supporting information

S1 Checklist Standards for Reporting Qualitative information (SRQR) checklist. (DOCX)

## List of abbreviations

CEmONC: Comprehensive emergency obstetric and neonatal care
CGTRH: Coast General Teaching and Referral Hospital
CI: Confidence interval
COVID-19: corona virus disease 2019
HDU: High dependency unit
HIC: High income country
LMIC: Low- and middle-income country
MO: Medical officer
PPE: Personal protective equipment
SARS-CoV-2: severe acute respiratory syndrome coronavirus 2
